# Awareness about Birth Registration in a Resettlement Colony of Delhi

**DOI:** 10.4103/0970-0218.51218

**Published:** 2009-04

**Authors:** Megha Gupta, Harsh Mahajan, P Lal

**Affiliations:** Department of Community Medicine, Maulana Azad Medical College, New Delhi - 110002, India

## Introduction

The UN Convention on the Rights of the Child is more than a decade old, but even today more than a third of all children are denied the right to an identity. Birth registration is the first legal document in which the name of the child gets entered along with the name of the parents. Globally, every year about 40 million children are born without being registered. Six out of ten unregistered newborns are in South and South-east Asia.(1) The registration of births and deaths in India was made compulsory under the Registration of Births and Deaths (RBD) Act, 1969. However, the current level of birth registration in India is 63.8% as per the latest 2005 provisional national estimates, which means that out of the estimated 26 million births taking place each year, approximately 9.4 million children (36.2%) go unregistered every year.(2) Taking these facts into consideration, this study was planned to assess the awareness about birth registration in an urban slum of Delhi.

## Materials and Methods

A cross-sectional study was conducted during the month of October 2004 in Basti Vikas Kendra, a slum area in Delhi, under the field practice area of the Department of Community Medicine, Maulana Azad Medical College. The slum has a population of 1,700 residing in 250 houses and the population under 5 years old is approximately 206. For the purpose of this study, a convenient sample of 83 houses was taken and every third house was selected by systematic random sampling. From every selected house, the information was taken from a single person preferably (mother, father, or anyone 18 years or above) after taking the consent of the individual.

Trained doctors collected information on the identification, socio-demographic profile, and awareness of the individual about birth registration (importance, ideal time, cost involved, etc.) using a structured questionnaire. Data was entered in an MS Excel™ spreadsheet and the findings are expressed in percentages.

## Results

Out of the 83 study subjects, 47 (56.6%) were males. The overall literacy rate was 72.3%, the percentage being more in the male respondents (76.5%) than the females (66.6). The mean age of the study participants was 36.3 years old. The females were mostly housewives (86.1%) while the males were involved in unskilled (42.5%), semi-skilled (4.2%), and skilled work (40.4%). The rest of the male subjects were unemployed (3.9%). The per capita monthly income (Rs) for the participants was in the range of 500-1299 (8.4%), 1300≤4000 (72.2%), and ≥ 4000 (19.4%).

The study findings in Table 1 show that there was overall agreement for the statement that informing the concerned authorities about any birth in the family is legally essential (63.8%). A total of 35 participants (42.2%) agreed with the statement that a birth certificate is the birth right of every child and 27 participants (32.5%) believed that the birth of a child can be registered even before deciding his/her name. More than half of the respondents i.e., 55.4% opined that the birth certificate is a legal document. The question on the cost involved in birth registration showed that 11 participants (13.3%) thought it did involve cost while the majority (56.5%) had no idea.

**Table 1:** Agreement of study subjects with certain statements regarding birth registration

Variables	n=83 (%)
School admission	53 (63.85)
Obtaining a ration card	50 (60.24)
Marriage registration	24 (28.91)
Obtaining a passport	23 (27.71)
Obtaining a driver's license	34 (40.96)
For insurance policies	33 (39.75)
For pension	32 (38.55)
For employment	44 (53.01)

Table 2 shows the awareness level of the study population regarding the use of a birth certificate as revealed by the following responses: School admission (63.9%), Ration card registration (60.2%), Marriage registration (28.9%), obtaining a passport (27.7%), obtaining a driver's license (41.0%), enrolling for insurance policies (39.8%), pension schemes (38.6%), and for employment (53.0%).

**Table 2:** Awareness of respondents regarding the various uses of a birth certificate

Variables	n=83 (%)
Informing the concerned authorities about birth is legally essential	53 (63.85)
A birth certificate is the right of every child	35 (42.16)
The birth can be registered even before selecting a name for the child	27 (32.53)
A birth certificate is a legal document	46 (55.42)
Birth registration involves cost	11 (13.25)

The ideal time of birth registration (within 21 days of birth) was mentioned correctly by only 3 (3.6%) of the study respondents while 30 participants (36.1%) knew where to get the birth registered. Of the total houses surveyed, there were 52 children under 5 years old. Out of these children, the births of 23 (44.2%) were registered and the same number of children were not registered. The information was not available for the rest. Out of the 23 births registered, the birth certificate was available for 17 (73.9%) children. The participants reported their source of information about birth registration as relatives/neighbors (33.7%), health personnel (27.7%), newspapers (15.6%), TV/Radio (12.0%), and hoardings (1.2%).

## Discussion

The study findings highlight that less than half of the births (44.2%) in the last 5 years were registered in this study area as compared with the national average of 63.8%. The findings are in contrast to the figures of more than 80% for the state of Delhi as reported in 2004.(3) The low awareness of the study respondents regarding the multifaceted use of birth certificates, the legality of the document, and the rights issue related to it can be attributed to a significant percentage of subjects being either illiterate (27.7%) or having received only primary education (44.6%). A large proportion of the study participants (67.5%) believed that the birth of a child cannot be registered before the identification of the child with his/her name. Some of the societies follow the custom of naming the child on a particular ceremony organized a few weeks after birth. This may be the reason for low birth registration especially in a country like ours where rituals still dominate the mindset of the people.

Around 13.3% of the respondents believed that registration of birth involves cost. This information could be the reason of one's experience of a delayed birth registration or maybe an involvement of an intermediary agent. The source of information was reported as relatives/neighbors by 33.7% of the participants and health personnel by 27.7% of the respondents. The other sources of media including the print media and the audio visual media seem to contribute minimally to their information. This points toward the need for increased IEC efforts through involvement of various effective channels of mass communication, which can repeatedly reinforce the concepts.

Though 63.9% of the subjects believed that registration of birth is compulsory, only 3.6% knew the correct time of getting a birth registered. In our study, births were registered for only 44.2% of the children under 5 years old, while the family possessed the birth certificate for 32.6% of the children under 5 years old. The figure is still better than that of the Multiple Indicator Survey that showed only 19% of the children younger than 5 years of age possess a birth certificate in India (Multiple Indicator Survey 2000).(3)

## Conclusion and Recommendations

This study highlights that the awareness regarding birth registration is inadequate among the study population, which is evident by the low percentage of births registered in the last five years. Efforts should be put towards the national target of achieving 100% registration of births by 2010 in accordance with the goal set by the National Population Policy, 2000.(4) The role of media has to be strengthened in the form of newspaper articles, reports, advertisements, etc. so that more people realize the importance of this legal document.
